# Non-Infectious Pneumonitis and Acute Respiratory Distress Syndrome in a Patient on Ustekinumab Treatment: Case Report and Literature Review

**DOI:** 10.3390/life14030394

**Published:** 2024-03-16

**Authors:** Valentina Cioffi, Giulia Di Napoli, Pierfrancesco Tozzi, Sabina Martelli, Katia Bruno, Andrea Longo, Helena Buso, Francesco Pugliese, Cinzia Milito

**Affiliations:** 1 Department of Translational and Precision Medicine, Sapienza University of Rome, 00161 Rome, Italy; valentina.cioffi94@gmail.com (V.C.); giulia.dinapoli@uniroma1.it (G.D.N.); 2 Department of Anesthesiology, Sapienza University, 00185 Rome, Italy; p.tozzi@policlinicoumberto1.it (P.T.); s.martelli@policlinicoumberto1.it (S.M.); katiabruno@me.com (K.B.); andrealongo495@gmail.com (A.L.); francesco.pugliese@uniroma1.it (F.P.); 3 Rare Diseases Referral Center, Internal Medicine 1, Department of Medicine (DIMED), AULSS2 Marca Trevigiana, Ca’ Foncello Hospital, University of Padova, 35124 Padova, Italy; buso.helena@gmail.com; 4 Department of Molecular Medicine, Sapienza University of Rome, 00161 Rome, Italy

**Keywords:** Ustekinumab, non-infectious pneumonitis, ARDS, ECMO, long-term treatment

## Abstract

Ustekinumab is a monoclonal antibody targeting the p40 subunit of IL-12 and IL-23, approved for treating psoriasis, psoriatic arthritis, and inflammatory bowel disease. Despite a remarkable success in treating chronic inflammatory conditions and a generally favorable safety profile, its role in inducing rare adverse events, such as interstitial pneumonia and acute respiratory distress syndrome (ARDS), remains largely uncharted. We report a case of a 66-year-old male patient treated with Ustekinumab for severe psoriasis who, after almost two years of treatment, developed dyspnea, asthenia, and fever progressing to non-infectious pneumonia and ARDS leading to ICU admission. Moreover, we conducted a literature review on Ustekinumab-associated pulmonary complications. Our case underscores the importance of appropriate and long-term clinical monitoring in patients on Ustekinumab treatment, particularly considering the potential lung complications. The possibility of non-infectious pneumonitis should be considered alongside infectious causes, facilitating prompt management in the case of negative infectious screening. Additionally, the severity of ARDS underscores the importance of timely recognition and proper management. Further investigations are recommended to investigate the immunological basis of Ustekinumab-induced ARDS for designing appropriate monitoring strategies.

## 1. Introduction

Ustekinumab, commercially known as Stelara, represents a significant advancement in the field of immunomodulatory therapy.

This monoclonal antibody is directed against the p40 subunit of IL-12 and IL-23 and was approved for the treatment of psoriasis, psoriatic arthritis, and inflammatory bowel disease [1].

It is usually a well-tolerated drug with a favorable safety profile despite some adverse effects reported, including interstitial pneumonia and even acute respiratory distress syndrome (ARDS) [2].

ARDS is a syndrome characterized by the sudden onset of hypoxic respiratory failure, associated with bilateral infiltrates found on lung imaging. ARDS can develop because of both direct and indirect injury to the lung, and early identification of the underlying etiology is critical for providing correct treatment [3]. 

Herein, we report a case involving a 66-year-old male treated with Ustekinumab for psoriasis who developed dyspnea, asthenia, and fever progressing to ARDS.

Moreover, we conducted a literature review to investigate the link between ARDS and Ustekinumab and to explain Ustekinumab’s mechanism of action [4].

## 2. Case Report

A 66-year-old male with a history of arterial hypertension, chronic HCV-related hepatitis, and successful treatment with Peg-IFN achieving sustained virologic response was admitted to the hospital for dyspnea, asthenia, and fever. He had been receiving treatment with Ustekinumab for nearly 2 years (45 mg subcutaneous every 12 weeks) for psoriasis, with his most recent dose administered 10 days prior to hospitalization, following the therapeutic regimen.

The patient had no reported anamnestic allergies and no relevant animal/environmental exposures related to job or hobbies and reported non-smoking status. Daily medications included valsartan 160 mg per day for hypertension. The clinical evaluation revealed an increase in inflammatory markers, and a chest HRCT scan highlighted right middle-to-lower lobe lung consolidation with areas of infiltration observed in the posterior superior left lobe. The arterial blood gas analysis revealed a pH of 7.48, partial pressure of carbon dioxide (pCO_2_) of 27, partial pressure of oxygen (pO_2_) of 75, and lactate level of 3.6, showing a complex acid–base imbalance with concurrent respiratory distress. In the suspicion of an infectious cause, also supported by a recent cruise, empirical antibiotic therapy was initiated and then upgraded with meropenem, linezolid, and azithromycin, along with an antifungal agent, caspofungin, and oxygen supplementation. The respiratory condition gradually deteriorated, requiring mechanical ventilation with a fraction of inspired oxygen (FiO_2_) of 50%, necessitating a shift to high-flow nasal cannula (HFNC) therapy at 60 L per minute (lpm) with 100% FiO_2_. While investigating potential causes of the observed respiratory distress, we systematically excluded several alternative pulmonary conditions.

Microbiological investigations were designed to rule out infectious etiologies, starting with bacterial exclusions. Tests covered respiratory pathogen testing, MRSA, urinary pneumococcal and legionella antigen detection, *Bordetella pertussis*, *Mycoplasma pneumoniae*, *Chlamydia pneumoniae*, and blood cultures. The detection of *Hemophilus influenzae* in a positive blood culture prompted an immediate shift in antibiotic therapy to teicoplanin and levofloxacin. Negative results were obtained for CMV DNA, SARS-CoV-2, and a range of other viral agents, including Adenovirus, Bocavirus, Metapneumovirus, Influenza A and B, Parainfluenza 1, 2, 3, 4, Rhinovirus, Enterovirus, and Respiratory Syncytial Virus (RSV). *Pneumocystis jirovecii* bacterial examination and galactomannan tests were negative, as well as cultures for typical and atypical mycobacteria.

All tests, including those for *Klebsiella pneumoniae*, were repeated at various intervals during the patient’s hospitalization to ensure a comprehensive evaluation.

Despite treatment, the patient’s clinical status and radiological findings worsened, requiring intensive care unit (ICU) admission. Due to rapid deterioration of the respiratory performance, the patient first needed non-invasive ventilation (NIV) cycles followed by intubation on day 11 due to worsening hypoxemia.

A thorough analysis to differentiate non-infectious causes was conducted. This involved excluding eosinophilic pneumonia through assessments of blood eosinophil counts and bronchoalveolar lavage (BAL) results. Connective tissue diseases, including rheumatoid arthritis and systemic lupus erythematosus, were considered and carefully ruled out based on clinical, serological, and imaging findings. Furthermore, we explored environmental exposures, ruling out potential interstitial lung diseases linked to toxins or occupational hazards. Notably, there was no history of occupational exposure contributing to interstitial pneumonia. The blood test showed positive anti-HCV antibodies, whereas HCV viral load was undetectable. Abdominal CT scan showed no evident signs of cirrhosis.

**Figure 1:**
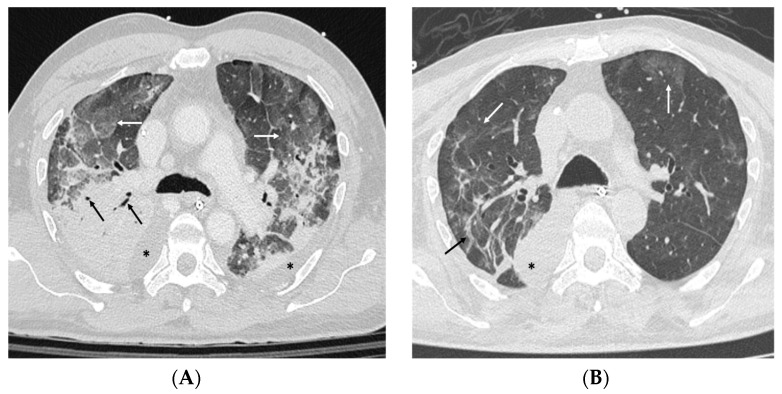
(**A**) (refers to the patient’s admission on the first day): Bilateral consolidations with air bronchogram (black arrows) and areas of ground glass opacities (white arrows). Bilateral Pleural effusion is also appreciable (asterisks). (**B**) (captures the patient’s status on the 26th day of hospitalization): Improvement of parenchymal abnormalities with some residual bands in the right lung (black arrows) and small bilateral areas of ground glass opacities (white arrows). Right pleural effusion is still present (asterisk).

A new chest HRCT scan showed bilateral pleural effusion, more evident on the right side, with atelectasis, as well as worsening of the inflammatory pattern with areas of lung consolidation associated with air bronchograms, together with confluent ground glass pattern involving all the free lung parenchyma (Figure 1A). Two days later, the worsening hypoxemia led to ARDS diagnosis, requiring invasive mechanical ventilation and veno-venous femoro-jugular extracorporeal membrane oxygenation (ECMO) support. The immunology consultant, based on the inflammatory pulmonary findings and on the negative microbiological workup, hypothesized a non-infectious pneumonia secondary to the monoclonal antibody Ustekinumab [5] that had been ongoing for two years. Thus, the patient was treated with high-dose steroid therapy (methylprednisolone 1 g/day for three days) and then prednisone 1 mg/kg/day starting from 80 mg/day. Prednisone was tapered by 20 mg every 4 days until 20 mg daily, with plans to continue this dose until outpatient follow up. We observed a steady improvement of respiratory status and blood gas exchange and the nearly complete resolution of lung inflammation.

Subsequently, after 14 days, the ECMO support was discontinued, and a tracheostomy was performed for gradually weaning from mechanical ventilation. A new HRCT scan showed bilateral reduction in inflammatory pattern and lung consolidation, even if some signs of pneumonia were still present in the superior and middle right lobe and in the superior left lobe (Figure 1B). Following Ustekinumab discontinuation, the patient’s psoriasis worsened, with a moderate increase in plaque size and erythema. The Psoriasis Area and Severity Index (PASI) reflected this exacerbation, with a score of 13 (Table 1).

The assessment emphasized the need for a careful balance between psoriasis management and critical care during the patient’s recovery. It led to the recommendation of alternative biologics, such as secukinumab, ixekizumab, or anti-TNF, considering the patient’s overall well-being in the intensive care context. Hemodynamic parameters returned to normal ranges without pharmacological support, chest imaging and clinical status improved, and the patient was discharged.

After the last administration, at the onset of the pulmonary picture, the drug was no longer administered because of the critical clinical situation. We discharged the patient with the instruction not to take it again and recommended an alternative biologic.

## 3. Discussion

Ustekinumab, a pivotal player in immunomodulatory therapy, has found success in treating various inflammatory conditions. Approved by the FDA for moderate to severe plaque psoriasis (Ps), its indication was first extended to psoriatic arthritis (PsA), and it has recently been approved for use in active Crohn’s disease (CD) and ulcerative colitis (UC).

This human monoclonal antibody modulates T-cell response acting against interleukin-12 (IL-12) and interleukin-23 (IL-23). IL-12 is involved in developing Th1 cells that secrete interferon-gamma (IFN-γ), heightening the cellular immune response against intracellular pathogens. In contrast, IL-23, a pro-inflammatory cytokine, sparks the differentiation and activation of Th17 cells, responding to extracellular pathogens, and is implicated in autoimmune diseases. Ustekinumab blocks the shared p40 subunit, inhibiting the interaction of these cytokines with the IL-12Rβ1 receptor found on NK cells and T cells, resulting in immune system downregulation [1,2,3,4,5,6].

Ustekinumab demonstrated a generally safe profile [7,8]. Common reactions encompass headache, bronchitis, abdominal pain, diarrhea, and fever. Adverse effects include hypersensitivity, infections, non-melanoma skin cancer, reversible posterior leukoencephalopathy syndrome, and pulmonary complications such as cryptogenic organizing pneumonia, interstitial or eosinophilic pneumonia, sarcoid-like reactions, and the rare manifestation of ARDS [9].

The reported association between IL-12/23p40 inhibitors and the development of interstitial lung disease (ILD), lung fibrosis, or ARDS raises the question about the possible underlying mechanisms. Studies indicate a dual role of IL-12 in lung fibrosis, with one study demonstrating its promotion of anti-fibrotic interferon-gamma (IFN-γ) production [10], while another suggests that IL-12p40 overproduction may contribute to fibrosis via macrophage accumulation [11].

Considering the patient’s extended exposure to Ustekinumab, we hypothesize that the drug’s inhibition of interleukin-12 (IL-12) and interleukin-23 (IL-23) pathways may have disrupted the delicate balance of pro-inflammatory and anti-inflammatory signals within the lung microenvironment. This dysregulated immune response could have primed the lung parenchyma to heightened sensitivity to inflammatory stimuli, potentially increasing the risk of ARDS onset. Therefore, our analysis suggests that prolonged Ustekinumab use may have predisposed the patient to ARDS by altering pulmonary immune homeostasis and facilitating an exaggerated inflammatory response. Before the spreading of Ustekinumab, different studies have primarily focused on the association between ARDS and other immunomodulatory agents, particularly tu-mor necrosis factor (TNF)-α inhibitors [12]. In recent years, several studies suggested a potential link between ustekinumab and pulmonary complications. 

Lee et al. reported the onset of ILD and Graves’ disease in a 68-year-old psoriatic patient under Ustekinumab treatment [13]. Kikuchi et al. reported Ustekinumab-induced interstitial pneumonia in two more psoriasis patients, both showing elevated KL-6 levels and ILD symptoms after treatment. Symptoms improved upon discontinuation of the medication, suggesting a potential association between the drug and the onset of interstitial pneumonia. KL-6 is a mucinous high-molecular-weight glycoprotein, expressed on type 2 pneumocytes, which is reported to be elevated in the serum and broncho-alveolar lavage fluid of patients with interstitial pneumonia. It has been suggested that monitoring KL-6 levels during Ustekinumab treatment might aid early detection of this adverse effect [14]. In a retrospective analysis by Brinker et al., twelve cases of non-infectious pneumonia associated with Ustekinumab were identified through the FDA Adverse Event Reporting System (FAERS). The temporal association was evident in all cases, with pulmonary symptoms emerging mostly after one to three doses of Ustekinumab and up to two years since the initiation of treatment. Additionally, positive dechallenge was observed in 58% of cases, including one positive re-challenge. For these reasons, discontinuation of Ustekinumab was suggested [15].

Furthermore, in a retrospective study by Miyagawa et al. involving 603 psoriasis patients treated with anti-IL-17/23 biologics, the incidence of drug-induced interstitial pneumonia (DIIP) was approximately 1.0%. Age, baseline KL-6 levels, and pre-existing interstitial pneumonia were highlighted as potential risk factors for DIIP associated with anti-IL-17/23 biologics. Ustekinumab, among other biologics, showed a mild course of DIIP with an average onset of 14 months after treatment initiation. Moreover, most cases demonstrated improvement after discontinuation of the causative biologics, emphasizing the significance of monitoring and early detection of DIIP during treatment [16].

In Marik’s paper, a 45-year-old woman with a history of psoriasis developed acute respiratory distress syndrome (ARDS) within days of receiving the first dose of Ustekinumab. Despite the authors’ observation that Ustekinumab is not associated with acute lung injury, our report, along with Despotes et al.’s insights and other relevant studies, challenges their hypothesis [17].

Despotes et al. reported a case of a 33-year-old man with Crohn’s disease who, after a positive response to Ustekinumab and a temporary discontinuation due to infectious concerns, resumed treatment for active Crohn’s disease. Notably, he developed acute hypoxic respiratory failure only two weeks after restarting Ustekinumab, requiring mechanical ventilation [18].

To note, he developed a respiratory failure requiring mechanical ventilation, mirroring our patient’s condition. This is, based on the data we found in the literature, the shortest period after which respiratory failure from Ustekinumab has been documented.

In our case report, a particular aspect is based on the prolonged two-year duration of Ustekinumab treatment before the onset of this rare pulmonary complication. As highlighted in the literature, most patients typically develop such complications in shorter time frames, even if few reports described the development of interstitial pneumonia after 2 years of treatment.

It is noteworthy that our patient’s two-year duration differs from typical cases. Importantly, our patient’s sole medication was valsartan, eliminating potential confounding factors present in other reports.

In summary, our experience and the literature review suggest a potential correlation between Ustekinumab use and severe pulmonary complications, such as non-infectious pneumonia and the development of ARDS, occurring from 2 weeks up to more than 2 years after treatment initiation.

Our case underscores the importance of appropriate and long-term clinical monitoring in patients on Ustekinumab treatment, particularly considering the potential lung complications. The severity of ARDS also emphasizes the need for timely recognition and proper management. Identifying potential risk factors, as highlighted by Miyagawa et al., and accurately excluding alternative causes, as emphasized by Brinker et al., are crucial steps in patients’ management [15,16].

Appropriate monitoring encompasses a comprehensive approach, including regular assessments for infectious causes, pulmonary function, and general well-being, as is standard for all biological therapies. This involves monitoring inflammatory markers, complete blood counts, vigilant observation for the onset of symptoms, and routine evaluations to ensure early detection of any potential adverse effects. Additionally, specific monitoring tailored to the patient’s medical history and pre-existing conditions may be incorporated to enhance the overall safety profile during Ustekinumab therapy. Additionally, for patients with psoriasis, monitoring the Psoriasis Area and Severity Index (PASI) is essential to track the severity and progression of the skin condition. For this reason, we suggest a tailored monitoring approach to patients considering individual variability in Ustekinumab response.

In our case, *H. influenzae* isolated from blood culture might have played a role as a trigger, complicating the clinical scenario. However, the response to steroids rather than to antibiotics underlines the contribution of Ustekinumab treatment to ARDS development. The timely use of steroids could be a critical option in mitigating the adverse respiratory effects of Ustekinumab. Moreover, the possibility to use early ECMO played a critical role in the management of our patient by limiting pulmonary stress due to aggressive and prolonged mechanical ventilation and allowing time for a more accurate differential diagnosis, particularly on the microbiological side [19].

Regarding the response to steroids in ARDS associated with Ustekinumab, it is important to clarify that a positive response is not exclusively related to Ustekinumab-induced pneumonitis. Steroids are commonly employed in the treatment of ARDS associated with infectious diseases, including COVID-19, and may elicit positive responses in various pneumonitis etiologies, such as hypersensitivity pneumonitis and eosinophilic pneumonitis. Notably, all these potential causes were thoroughly excluded in our case, ensuring a more accurate representation of the clinical context and avoiding potential misinterpretations. In addition to the specific case presented, it is important to consider the broader context of potential risk factors for non-infectious pneumonia. These include the use of medications that can damage the lungs, inhalation of material caused by aspiration, exposure to irritants or allergens triggering an immune response, and exposure to radiation. Recognizing and addressing these potential risk factors is crucial in clinical practice when evaluating patients presenting with respiratory symptoms, particularly in the absence of infectious etiologies. By identifying and addressing these factors, clinicians can provide more targeted and effective management for patients with non-infectious pneumonia.

In conclusion, in the presence of respiratory failure during Ustekinumab treatment, the possibility of a non-infectious pneumonitis should be considered while ruling out the possible infectious causes, facilitating prompt management in the case of negative infectious screening [5]. Further investigations are recommended to unravel the immunological basis of Ustekinumab-induced ARDS, providing a comprehensive understanding of mechanisms and potential risk factors to design appropriate monitoring strategies.

## Figures and Tables

**Table 1:** Clinical and laboratory parameters and medications during hospitalization.

Day of Hospitalization	Respiratory Status	Pulmonary Changes	Lab Data	Medication Dosages	Other Clinical Data
1	Tachypnoeic in VMK, FiO_2_ 50%. shifted to HFNC 60 lpm-100%,	Basal consolidation right lung.	pH 7.48, pCO_2_ 27, pO_2_ 75, lactate 3.6 WBC 4000 cells/mmc CRP: 516,600 mg/L	Merrem 1 g × 3, Linezolid 600 mg × 2, Azithromycin 500 mg.	Diarrhea, consolidation on chest X-ray, initiated oxygen therapy.
2	HFNC 60 lpm, FiO_2_ 80%.	Bilateral opacities.	pH 7.45, pCO_2_ 33, pO_2_ 247, lactate 3.2, P/F 274	Targosid (replaced Linezolid), Cancidas 50 mg/die Aerosol therapy (Fluibron + Broncovaleas).	PASI: 0.
3	HFNC 60 lpm, FiO_2_ 80%.	Stable.	pH 7.43 pCO_2_ 38, pO_2_ 193, Lac 1.5, P/F 276 CRP: 345,000 mg/L	Levoxacin 750 mg/die.	*H. Influentiae* in blood cultures.
5	HFNC 60 lpm, FiO_2_ 100%.		pH 7.42 pO_2_ 177, pCO_2_ 36, Lac 1.1 CRP 272,000 WBC 12.000 cells/mmc	Ongoing with same treatment.	
6	HFNC 60 lpm, FiO_2_ 90% Started NIV cycles (PS 10, PEEP 8, FiO_2_ 90%).		pH 7.41 pO_2_ 101, pCO_2_ 34, Lac 1.1, P/F 112 WBC 16,200 cells/mmc		Sterile placement of CVC in the right internal jugular vein with Seldinger-guided ultrasound technique, smooth procedure. Chest X-ray for control.
7	HFNC 60 lpm, FiO_2_ 90% Placed CPAP helmet PEEP 8 at 100% FiO_2_ for a two-hour cycle, twice a day.	Chest X-ray unchanged.	Ph 7.46 pO_2_ 90, pCO_2_ 38, Lac 1.6, P/F 100		Blood cultures performed on febrile spike.
8	Eupnoic in HFNC 60 lpm FiO_2_ 95%. NIV initiated for the night (Ps 8 Peep 8 FiO_2_ 90%).	Mild clearing of hypodiaphania in the right middle-basal area.	pH 7.48, pCO_2_ 37, pO_2_ 87, lactate 1.5, P/F 92	Linezolid added after consulting infectious disease specialist.	Thoracentesis with aspiration of 1500 cc sierous fluid; microbiological tests performed.
9	Eupnoic in HFNC 60 lpm-100%. NIV-Helmet PS 12 PEEP 8 FiO_2_ 100%.	Chest X-ray unchanged.	pH 7.48, pCO_2_ 42, pO_2_ 89, Lac 1.5	Clexane 8000 UI sc a day.	
10	Tachypnoeic in NIV helmet (Ps 12 PEEP 8 FiO_2_ 90%), increased Ps to 14. Switched to HFNC 60 L/min, FiO_2_ 100%. NIV helmet applied (Ps 16 PEEP 8 FiO_2_ 100%).		pH 7.42, pO_2_ 191, pCO_2_ 49, Lac 1.5, P/F 191 pH 7.42, pCO_2_ 49, pO_2_ 105, lactate 1.7 pH 7.40, pO_2_ 69, pCO_2_ 50, Lac 1.7 WBC and PCR slightly reduced		
11	Tachypneic in NIV helmet (PS 16, PEEP 8, FiO_2_ 100). Intubation performed with videolaryngoscope (8 mm tube); connected to ventilator in SIMV mode Vt 550 mL RR 16 PEEP 10 FiO_2_ 80%.	Chest X-ray control.	pH 7.42, pCO_2_ 52, pO_2_ 164, LAC 1.6, P/F 164 pH 7.40, pO_2_ 122, pCO_2_ 37, Lac 1.2, P/F 244	Sedation with propofol and disufen, noradrenaline (IC: 14 mL/h).	Deterioration of clinical status; performed FBS, sent BAL samples to microbiology and virology.
12	Critical condition. Intubated and ventilated in SIMV (Vt 500 RR 16 PEEP 10 FiO_2_ 80%) ECMO VV started (2320 rpm, BF 3, FGF 4, FiDO_2_ 100%).	Chest/abdomen CT scan performed.	pH 7.43 pCO_2_ 53 pO_2_ 79 LAC 2.4 P/F 99 pH 7.50 pCO_2_ 42 pO_2_ 88 lactate 2.9 pH 7.40, pCO_2_ 53, pO_2_ 158, lactates 2.2	Hemodynamics supported by noradrenaline in IC at 10 mL/h (4 mg:50 mL SF). Bactrim added to therapy.	
13	Ventilated in SIMV (VT 330, RR 10, PEEP 8, FiO_2_ 100) with vvECMO (BF 3, FGF 4, FiDO_2_ 100).	Chest X-ray FBS control.	EGA: pH 7.47, PaCO_2_ 54, PaO_2_ 84, Lactate 2.9	Propofol, sufentanil, midazolam, noradrenaline, Amiodarone, Furosemide, ATIII, Heparin, PFC.	Hemodynamically unstable.
14	Ventilated in SIMV (VT 330, RR 10, PEEP 8, FiO_2_ 80) with vvECMO (BF 3, FGF 3, FiDO_2_ 100). Increased BF to 3.5 L/min.	Chest X-ray shows persistent bilateral parenchymal opacities.	pH 7.47, pCO_2_ 57, pO_2_ 80, Lac 3.2. pH 7.52, pCO_2_ 46, P/F 78, Lac 3.4, HCO3 35	Hemodynamically supported with noradrenaline 0.35 mcg/kg/min (increased overnight 0.42 mcg/kg/min. Corrected antithrombin. Ongoing heparin infusion at 1.9 mL/h.	
15	Intubated and connected to VM in SIMV (VT 330, RR 10, PEEP 8, FiO_2_ 80, Pplat 20, Cd 21) with vvECMO (BF 3, FGF 3.5, FiDO_2_ 100).		pH 7.46, pO_2_ 89, pCO_2_ 57, Lac 3.3,		
16	vvECMO continued (2465 RPM BF 3.25 FGF 3 FiDO_2_ 100%).	FBS performed, abundant collose secretions aspirated from both hemispheres. Chest X-ray unchanged.	pH 7.41, paCO_2_ 56, paO_2_ 98, Lac 2.9, CRP 98,600, WBC 10,280 cells/mmc	Solumedrol 1 g/day for 3 days after immunological consult. Hemodynamically supported by noradrenaline in ICU at 3 mL/h of 4 mg in SF 50 mL mcg/kg/min.	sinus bradycardia at a medium frequency of 48 bpm. Immunological consult.
20	vvECMO continued (2465 RPM BF 3.25 FGF 3 FiDO_2_ 100%).			Solumedrol 80 mg/day.	PASI: 7.
24	ECMO support removed; Ventilation modified: TV 600 mL, RR 12, PEEP 8, FiO_2_ 60%.		pH 7.44, pCO_2_ 61, pO_2_ 94, lactate 1.7, P/F 157. pH 7.50, pCO_2_ 50, pO_2_ 107, lactate 1.7, P/F 214	Noradrenaline in ICU at 5 mL/h (4 mg:50 mL SF). Solumedrol 60 mg/day.	
26	After curarization, under fibrobronchoscopic view, percutaneous tracheostomy is performed using the Griggs technique, placing a size 8 cannula, FBS is performed: thick secretions are aspirated (right > left).	Chest CT performed.	pH 7.56; pCO_2_ 48, pO_2_ 105, lactate 1.3; Hb 9.7, P/F 210, WBC 20,620 cells/mmc		Sedation suspended.
30	Tracheostomized and ventilated in PSV (PS 14, PEEP 5, FiO_2_ 40%). Placement of HFOT started at 60 lpm, gradually reduced to 40%.		pH 7.51, pCO_2_ 45, pO_2_ 115, lactate 1.6, P/F 288. WBC 19,890 cells/mmc	Valsartan 160 mg reintroduced in therapy.	PASI 13.
44			pH 7.45, paO_2_ 97, paCO_2_ 36, Lac 1.1		PASI 16 Patient discharged.

FiO_2_: fraction of inspired oxygen; EGA: arterial blood gas analysis; pCO_2_: partial pressure of carbon dioxide; pO_2_: partial pressure of oxygen; P/F: ratio of PaO_2_ to FiO_2_; Lac: lactate levels; PSV: pressure support ventilation; PEEP: positive end-expiratory pressure; PS: pressure support; WBC: white blood cell count; Cancidas: Caspofungin; CRP: C-reactive protein.
